# The Bigger, the Better: Genetic and Phenotypic Analysis of Fruit Size in Sweet (*Prunus avium* L.) and Sour Cherry (*Prunus cerasus* L.) Germplasm

**DOI:** 10.3390/plants15060856

**Published:** 2026-03-10

**Authors:** Sámuel Szilágyi, Francesco Desiderio, Balázs Marton, Piroska Mohay, Alejandro Therese Navarro, Zsuzsanna Békefi

**Affiliations:** 1 Research Centre for Fruit Growing, Hungarian University of Agriculture and Life Sciences (MATE), Elvira major, 2030 Érd, Hungary; 2 National Research Council of Italy, Institute of Biosciences and Bioresources, Corso Calatafimi, 414, 90129 Palermo, Italy; 3 Plant Breeding Research Group, Wageningen University & Research, Radix, Droevendaalsesteeg 1, 6708 PB Wageningen, The Netherlands

**Keywords:** *Prunus avium*, *Prunus cerasus*, fruit size, SSR, germplasm

## Abstract

Fruit size and weight are valuable characteristics for cherry breeders, mainly because of their higher market price. Several molecular markers have been developed in recent years and have been correlated with fruit weight. In cherries, *FW_G2a* was identified as a promising hotspot for fruit size and weight characteristics. Two markers flanking that region were taken into consideration in this study. The local sweet and sour cherry collection in Érd, Hungary, was analyzed using molecular markers to identify possible correlations between the markers and phenotypes. The duration of phenotypic observations varied from 3 years in sour cherry to 5 years in sweet cherry. In our study, we observed correlations between fruit size and weight and the molecular markers of our germplasm collection. We confirmed the previously published association of haplotypes 190–255 and 192–233 with large and small fruit size, respectively, in sweet cherry. Individual alleles of both markers were identified, showing moderate to strong correlations with large and small fruit size in sweet cherry. In tetraploid sour cherry, a higher number of unique allelic combinations were found due to the higher level of ploidy compared to sweet cherry. Individual alleles were detected with moderate positive correlations with fruit size, while one allele showed a strong negative correlation with fruit dimension-related traits. These markers were found to be useful for the characterization of fruit size characteristics, for population selection and for the differentiation of the Hungarian germplasm collection.

## 1. Introduction

### 1.1. Sweet and Sour Cherry Origin and Production

Sweet cherry (*Prunus avium* L.) and sour cherry (*Prunus cerasus* L.) are economically important stone fruits bred and propagated mainly for fruit production. The area of origin of sweet and sour cherry is commonly agreed to be between Eastern Europe and near West Asia [1,2]. In particular, sweet cherry originates from the territory around the Caspian and Black Seas and is considered either indigenous to the region or introduced during the Neolithic and Bronze Age (5500–4000 BCE) [3,4]. Sour cherry is instead presumably native to the region between the Caspian Sea and Northwest and Central Europe [5]. Being native to the European territory, it is only natural that those stone fruits have been bred for a long time in Europe and West Asia, becoming a traditional staple food in the local culture. Sweet cherry international production and market are dominated by several countries, such as Turkey, USA, Chile, Uzbekistan, Iran, Spain, Italy, Greece, Ukraine and Syria, with a world production of around 2.6 million tons in 2020, with Hungarian production in the range of 12 thousand tons/year [6,7]. Sour cherry is instead cultivated mainly in Russia, Ukraine, Turkey, Poland, Serbia, Iran, USA, Uzbekistan and Hungary, with a production of 1.4 million tons in 2020, Hungary in particular being among the top ten producers worldwide, with around 60 thousand tons/year [8]. Both fruits can be consumed fresh or preserved, such as jams, juices, and derived alcoholic beverages. Consumers’ preferences regarding sweet cherry suggest that in Europe the main priority is for firm red or dark red fruit skin color, with a general appreciation for bigger fruit. Sour cherry fresh consumption is limited, but generally a balanced acidity-to-sweetness ratio is appreciated by consumers [9]. For this reason, in recent years, breeding has focused on fruit size, firmness and fruit skin color for the commercialization of superior new varieties.

### 1.2. Sweet and Sour Cherry Genetics and Fruit Size Genotyping

Sweet cherry (*Prunus avium* L.) is diploid (2n = 2x = 16) with a relatively small genome (338 Mb). Sour cherry (*Prunus cerasus* L.) has instead been recently identified as a natural hybrid between sweet cherry (*Prunus avium* L.) and Mongolian cherry (*Prunus fruticosa* Pall.) and is an allotetraploid (2n = 4x = 32) species with a 599 Mb genome [10]. Both sweet and sour cherry genomes have been sequenced in recent years, giving a clearer picture of gene expression and functionality, such as the development of a 6K SNP (Single Nucleotid Polymorphism) array for sweet and sour cherry, long-read and Hi-C sequencing and scaffolding in sweet cherry, sequencing of the sour cherry genome and transcriptome, and whole-genome resequencing of sweet cherry [11,12,13,14]. Molecular marker development based on known genomes provided the opportunity to identify valuable trait-related markers. Regarding sweet cherry genetic resources, a comprehensive study was performed including 314 accessions across Europe, where high diversity of this material was demonstrated by SSR (Simple Sequence Repeat) marker analysis [15]. Among 14 SSR loci analyzed, BPPCT034 proved to be the most polymorphic, having 15 different alleles and an H_0_ value of 0.95. The CPSCT038 marker was not as polymorphic, and six alleles were represented in the genotypes studied.

The first study on cherry fruit size genetics was performed by D. Wang and colleagues [16], where they identified QTLs (Quantitative Trait Loci) on linkage group 2 and also on chromosome 1 of ʽÉrdi bőtermő’.

Most studies identified a hotspot region of the sweet cherry genome on linkage group 2 that proved to be the most associated with the fruit size phenotype [17,18]. In a study that involved four progenies and 23 cultivars, the most relevant QTL was FW_G2a on LG2, flanked by CPSCT038 and BPPCT034 markers [19]. This chromosome region appeared to hold genes of other important traits such as flowering time [20] and fruit firmness [21]. Molecular analysis on sweet cherry fruit size with ROSBREED 6K cherry SNP array v1 on LG2 identified haploblocks for further selection [22].

Other linkage groups also appeared to play a role in fruit size. Campoy and colleagues [21] identified regions in all eight linkage groups that have various effects on fruit size. Recently, Holušová and colleagues [23] identified markers involved in cherry fruit size on LG 1, 4 and 8 by genome-wide association studies (GWAS) analyzing more than 1.7 million SNPs. Similarly, McCord and colleagues [24] used GWAS in a germplasm population using the cherry 6+9 K SNP array. In this study, SNP markers were found in each linkage group associated with fruit size.

Available information on fruit size marker analysis of sour cherry is sparse. De Franceschi and colleagues [18] developed markers associated with loci linked to fruit weight on different linkage groups. Certain alleles on LG2 showed a strong association with fruit weight parameters, as well as on LG6, which was not the case in sweet cherry.

Using the available information, our main goal was to extend the utilization of markers associated with fruit size to further sweet and sour cherry accessions. Our previous research conducted on sweet cherry [25] gave promising results, so we decided to continue our analysis with the two simple sequence repeat (SSR) markers, CPSCT038 and BPPCT034 [26,27], focusing on the *FW_G2a* locus on LG2. Highly polymorphic SSR markers provide high information content at a specific locus. In our case, these markers were selected since they correlate with large- and small-fruited genotypes in sweet cherry, as they are both located on the *FW_G2a* locus on LG2, flanking the *PavCNR12* (Cell Number Regulator) gene. The *PavCNR12* gene co-localizes with the peak of the most important QTL for fruit size, and it is hypothesized to control fruit size in sweet cherry [18,25]. We focused our research on the germplasm collection of sweet and sour cherries available in Érd, Elvira major (15 km from Budapest), hosted by the Hungarian University of Agriculture and Life Sciences (Magyar Agrár- és Élettudományi Egyetem, MATE), Horticultural Institute, Research Centre for Fruit Growing. The germplasm collection was established in the 1950s and currently hosts more than 200 local sweet and sour cherry accessions each, where Hungarian local cultivars and landraces, as well as foreign cultivars, are preserved ex situ and analyzed [28]. The availability of plant material allows researchers to access information regarding superior alleles, thereby determining genetic improvement to raise the bar with each successive generation of new cultivar release [29,30].

Our main objective was to analyze and identify possible candidate material for pre-breeding and breeding selection and integration in future commercial breeding using molecular markers correlating with fruit size characteristics and to select parental lines with high breeding potential in the germplasm collection, as well as to enable early screening of progenies and selection for different fruit sizes in hybrid populations.

## 2. Results

### 2.1. Sweet Cherry Phenotypic Analysis

**Figure 1:**
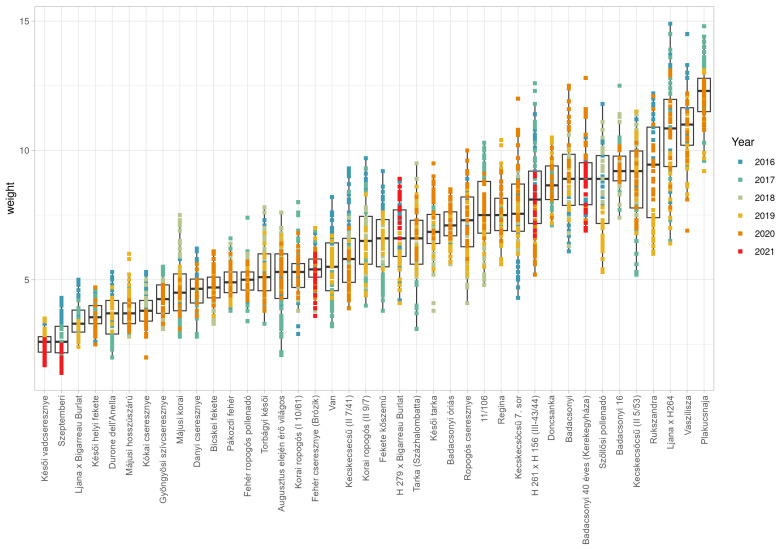
Sweet cherry weight (g) for each accession measured between 2016 and 2021. Boxplots represent individual measurements in each year according to color coding. Blue indicates measurements in 2016, light blue measurements in 2017, grey measurements in 2018, yellow measurements in 2019, orange measurements in 2020 and red measurements in 2021.

The forty sweet cherry accessions analyzed between 2016 and 2021 showed differences in fruit size and fruit weight. The smallest recorded fruit size was observed in ‘Késői vadcseresznye’, where fruit diameter (16.07 mm), length (15.90 mm) and thickness (14.10 mm) were the smallest across the years. On the contrary, the accession ‘Plakucsnaja’ had the largest fruit, with fruit diameter 28.82 mm, length 25.51 mm and thickness 24.35 mm (Table S1). Great variation in fruit size was observed between accessions, where the smallest fruit was 2.55 g in ‘Késői vadcseresznye’ and the largest fruit was 12.18 g in ‘Plakucsnaja’ (Figure 1).

### 2.2. Sour Cherry Phenotypic Analysis

**Figure 2:**
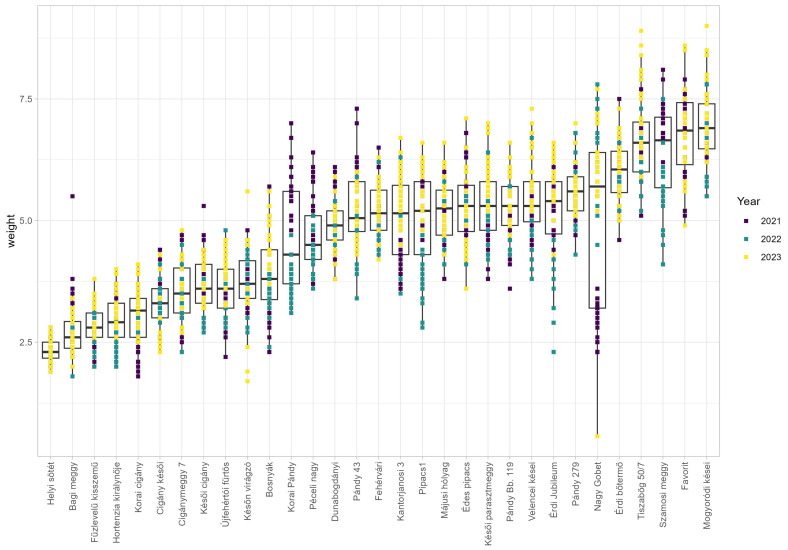
Sour cherry weight (g) for each accession measured between 2021 and 2023. Boxplots represent individual measurements in each year according to color coding. Purple indicates measurements in 2021, aquamarine measurements in 2022 and yellow measurements in 2023.

In the case of sour cherry, 31 individual accessions were analyzed in our study. Three consecutive years of phenotyping were performed between 2021 and 2023 for fruit size and fruit weight. The accession ‘Helyi sötét’ showed the smallest fruit diameter (14.44 mm), length (12.43 mm) and thickness (12.07 mm), while the largest accession was ‘Mogyoródi kései’ for fruit diameter (22.48 mm), length (18.91 mm) and thickness (18.63 mm) (Table S2). Observed fruit weight varied between 2.31 g (‘Helyi sötét’) and 6.93 g (‘Mogyoródi kései’) among the assessed accessions (Figure 2).

### 2.3. Sweet Cherry Genetic Analysis and Association with Phenotype Data

**Figure 3:**
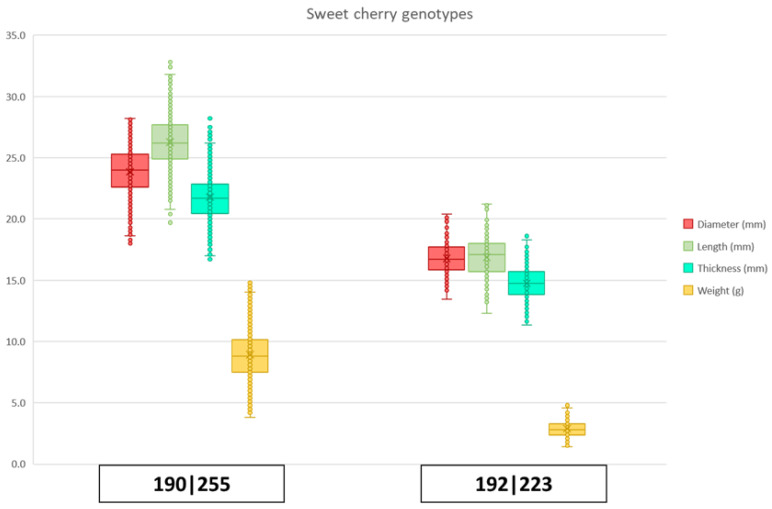
Two main genotypes observed in sweet cherry cultivars for CPSCT038 and BPPCT034. Indicated are the possible haplotypes 190–255 and 192–223. In red are the measurements for diameter (mm), in green are the measurements of length (mm), in aquamarine are the measurements for thickness (mm) and in yellow are the measurements for weight (g).

Molecular markers tested displayed polymorphism in sweet cherry, where nine alleles were observed for BPPCT034, with lengths between 222 and 257 bp, and four alleles were observed in the case of CPSCT038, spanning 190 to 204 bp (Table 1 and Table S1). The main observed genotypes of the closely linked markers CPST038 and BBPCT were 192/192 and 223/223 (‘Késői vadcseresznye’, ‘Szeptemberi’, ‘Ljana × Bigarreau Burlat’), 192/204 and 223/235 (‘Bicskei fekete’, ’Májusi korai’, ‘Torbágyi késői’), 190/190 and 257/257 (‘Kókai cseresznye’, ‘Kecskecsecsű’, ‘Korai ropogós’, ‘Rukszandra’), and 190/190 and 255/255 (‘Fekete kőszemű’, ‘Kecskecsöcsű’, ‘H 261 × H 156’, ‘Doncsanka’, ‘Kerekegyháza’, ‘Badacsonyi’, ‘Badacsonyi 16’, ‘Ljana × H264’ and ‘Plakucsnaja’). Possible haplotypes could also be identified, as indicated in Table S1. Despite unequal variances observed, one-way ANOVA differentiated genotypes into 10 significantly different groups based on their fruit weight. The group with the most accessions carried the CPST038_190_ and BPPCT034_255_ alleles in homozygous form and had a mean fruit weight of 8.89 g. The accessions with the smallest fruits had the alleles CPST038_192_ and BPPCT034_223_, both in homozygous form (Figure 3).

**Figure 4:**
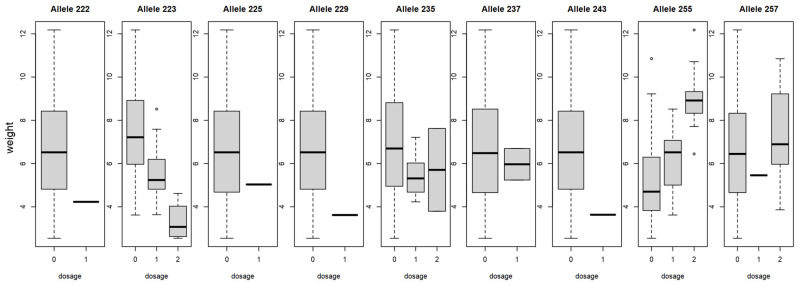
Dosage effect of BPPCT034 alleles on fruit weight (g) in sweet cherry. Boxplot dosage represents how many alleles are present (1, 2) or absent (0).

**Figure 5:**
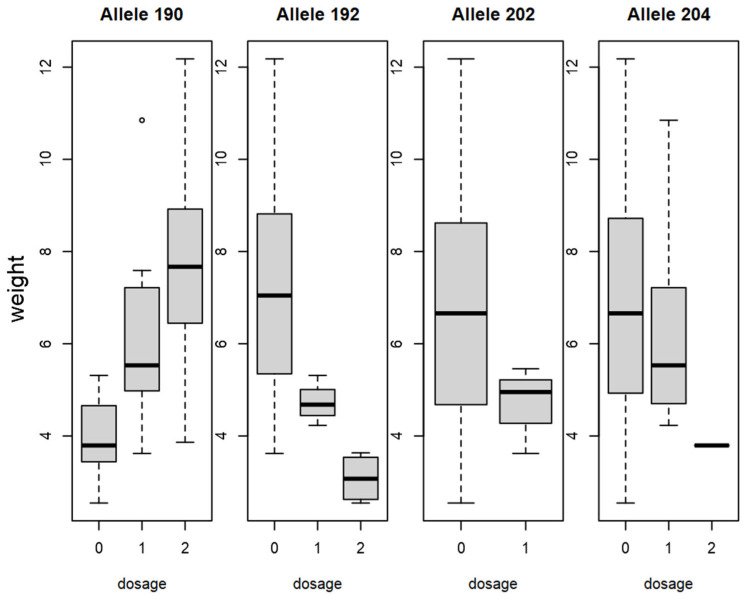
Dosage effect of CPSCT038 alleles on fruit weight (g) in sweet cherry. Boxplot dosage represents how many alleles are present (1, 2) or absent (0).

Pearson’s correlation matrix showed a correlation between alleles and phenotypes (Table S3): Between fruit size (length, diameter, thickness), weight and the two alleles, CPSCT038_190_ and BPPCT034_255_, a positive correlation was observed. Furthermore, BPPCT034_223_ negatively correlated with fruit size and weight. Pearson’s correlation indicates a positive correlation between CPSCT038_190_ and diameter (r(3066) = 0.55, *p* < 0.01), length (r(3066) = 0.54, *p* < 0.01), thickness and weight (r(3066) = 0.51, *p* < 0.01 each). CPSCT038_192_ instead indicated a negative correlation for diameter (r(3066) = −0.49, *p* < 0.01), length (r(3066) = −0.49, *p* < 0.01), thickness (r(3066) = −0.44, *p* < 0.01) and weight (r(3066) = −0.45, *p* < 0.01). BPPCT034_255_ showed a positive correlation with diameter (r(3066) = 0.45, *p* < 0.01), length (r(3066) = 0.45, *p* < 0.01), thickness (r(3066) = 0.44, *p* < 0.01) and weight (r(3066) = 0.44, *p* < 0.01), while a negative correlation was observed between BPPCT034_223_ and diameter (r(3066) = −0.40, *p* < 0.01), length (r(3066) = −0.53, *p* < 0.01), thickness (r(3066) = −0.44, *p* < 0.01) and weight (r(3066) = −0.43, *p* < 0.01). An allelic dosage effect could be observed in the case of several alleles for fruit weight (Figure 4 and Figure 5). The boxplot of allele BPPCT034_255_ confirms that, in homozygous form, this allele corresponds to higher fruit weight, while allele BPPCT034_223_ in homozygous form contributes to smaller fruit. As for the marker CPSCT038, if the allele CPSCT038_190_ is not present, it suggests a smaller fruit size, and the presence of allele CPSCT038_192_ in homozygous form also contributes to a decrease in fruit weight.

### 2.4. Sour Cherry Genetic Analysis and Association with Phenotype Data

**Figure 6:**
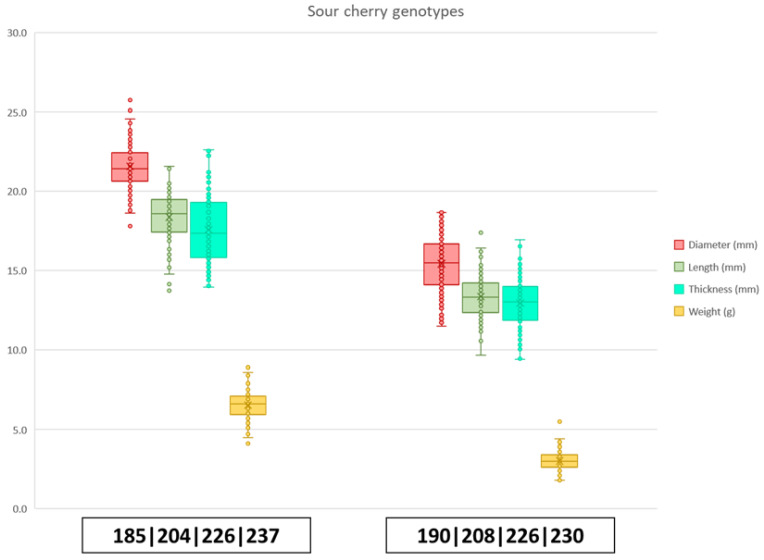
Boxplot for two distinct sour cherry genotypes 185/185/185/185–204/204/226/237 and 190/190/190/190–208/208/226/230. In red are the values for diameter (mm), in green for length (mm), in aquamarine for thickness (mm) and in yellow for weight (g).

Two SSR markers were tested on the 31 accessions, where similar allelic polymorphism was observed, as in sweet cherry. Allele sizes of BPPCT034 ranged between 204 bp and 251 bp, while CPSCT038 allele sizes ranged between 185 bp and 204 bp. In total, 10 alleles were found for BPPCT034 and three for CPSCT038. Furthermore, a variety of genotypes was observed due to the high polymorphism and the higher level of ploidy in tetraploid sour cherry. In total, 21 unique allelic combinations (genotypes) were detected (Figure 6 and Table S2). The observed groups based on the alleles of CPSCT038 and BPPCT034 markers, respectively, were composed of accessions ‘Bagi meggy’, ‘Korai cigány’ and ‘Cigány késői’ (190/190/190/190–208/208/226/230); ‘Dunabogdányi’, ‘Májusi hólyag’ and ‘Édes pipacs’ (185/185/204/204–204/204/226/237); ‘Késői parasztmeggy’ and ‘Velencei kései’ (185/185/204/204–226/226/237/237); ‘Péceli nagy’, ‘Pándy Bb. 119’, ‘Pándy 43’, ‘Nagy Gobet’ and ‘Pándy 279’ (190/190/204/204–204/204/226/237); ‘Szamosi meggy’ and ‘Tiszabög 50/7’ (185/185/185/185–204/204/226/237), while the other observed genotypes were all unique, as shown in Table S2. According to one-way ANOVA, seven significantly different groups were formed based on the fruit weight of the 21 genotypes, despite the unequal variances observed.

**Figure 7:**
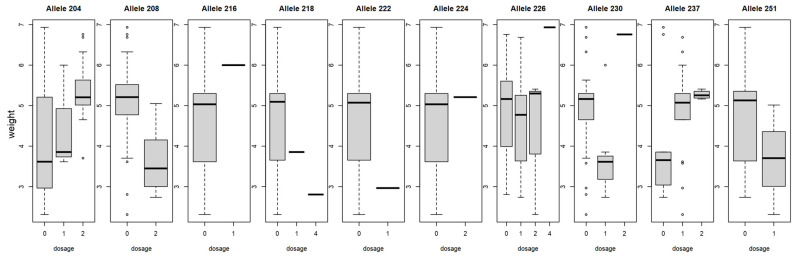
Dosage effect of BPPCT034 alleles on fruit weight (g) in sour cherry. Boxplot dosage represents how many alleles are present (1–4) or absent (0).

**Figure 8:**
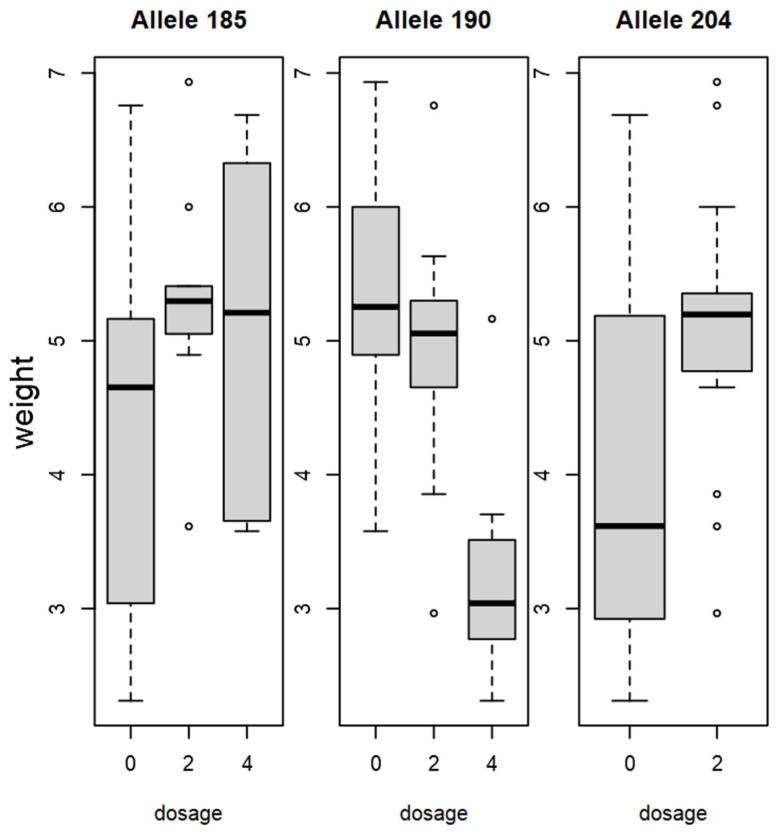
Dosage effect of CPSCT038 alleles on fruit weight (g) in sour cherry. Boxplot dosage represents how many alleles are present (1–4) or absent (0).

When genotype data were compared to the phenotype using Pearson’s correlation, BPPCT034 and CPSCT038 alleles showed correlations with fruit size and weight characteristics, although significant results for marker BPPCT034 were discovered only for a few alleles. BPPCT034_204_ and BPPCT034_237_ alleles both showed a positive correlation with each physical characteristic, although a significant correlation in every case could be detected only for the former (Table S4). CPSCT038_190_ showed a negative correlation with diameter (r(1748) = −0.35, *p* < 0.05), length (r(1748) = −0.35, *p* < 0.05), thickness (r(1748) = −0.32, *p* < 0.05) and weight (r(1748) = −0.039, *p* < 0.05), while CPSCT038_185_ and CPSCT038_204_ both showed significant positive correlations with fruit size. BPPCT034_208_ showed a negative correlation with fruit diameter (r(1748) = −0.5, *p* < 0.01), length (r(1748) = −0.55, *p* < 0.01), thickness (r(1748) = −0.54, *p* < 0.01) and weight (r(1748) =−0.51, *p* < 0.01). BPPCT034_204_ instead showed a positive correlation with diameter (r(1748) = 0.49, *p* <0.01), length (r(1748) = 0.51, *p* < 0.01), thickness (r(1748) = 0.51, *p* < 0.01) and weight (r(1748) = 0.46, *p* < 0.01). A dosage effect of certain alleles could be observed on fruit weight. The presence of allele BPPCT034_237_ in one or two copies contributed to a higher fruit weight, while the absence of this specific allele led to smaller fruit size in our findings. As for allele BPPCT034_208_, a homozygous form was found in sour cherries with smaller fruit size. Accessions carrying the CPSCT_190_ allele in two or four copies were found to have smaller fruits, compared to the ones where it was absent. A higher fruit weight was associated with the allele CPSCT_192_ if it was detected in homozygous form (Figure 7 and Figure 8).

### 2.5. Principal Component Analysis for Sweet and Sour Cherry

**Figure 9:**
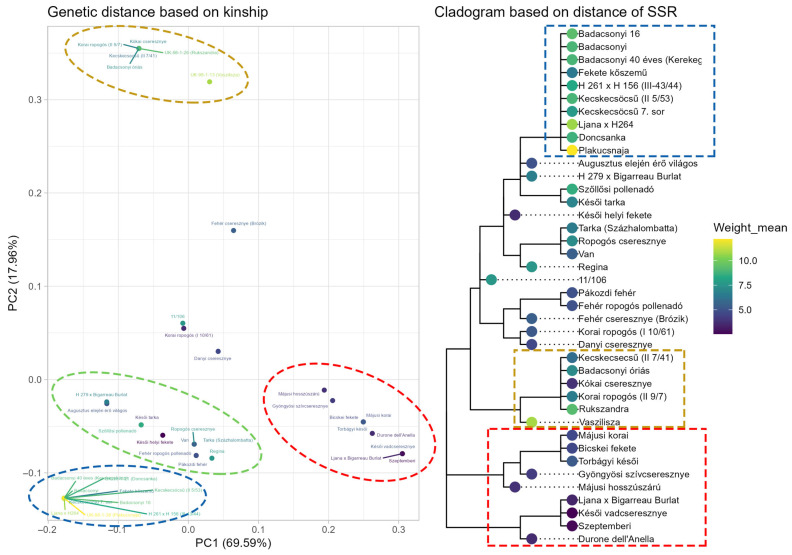
Genetic distance based on kinship in sweet cherry. Principal component analysis based on BPPCT034 and CPSCT038 indicates four distinct groups in dark yellow, red, green and blue.

Principal component analysis (PCA) for sweet and sour cherry was performed using the two SSR markers BPPCT034 and CPSCT038, and weight, which allowed observation of distinct groups based on the molecular markers’ size and combinations. In the case of sweet cherry, four distinct groups were identified based on alleles of the analyzed SSR markers, where weight varied between small (4 g), small-to-medium (6 g), medium (7.1 g) and large fruits (8.9 g) (Figure 9). Accessions with small fruit size, grouping together, consistently carried alleles BPPCT034_223_ and BPPCT034_235_, in one or two copies. The CPSCT038_192_ allele, in either homozygous or heterozygous form, was present only among these accessions. Accessions that visually clustered together and had high fruit weight carried the alleles CPSCT038_190_ and BBPCT034_255_ exclusively in homozygous form. The small-to-medium-sized fruit group visually clustered together well on the PCA plot; however, it did not separate clearly on the cladogram.

**Figure 10:**
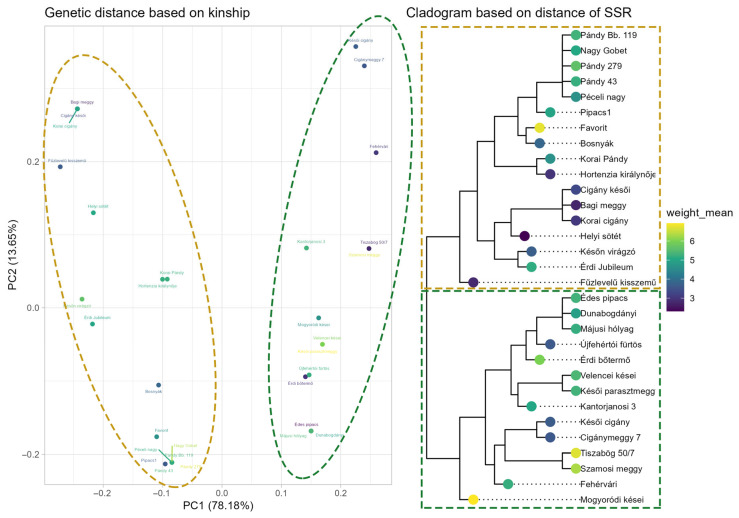
Genetic distance based on kinship in sour cherry. Principal component analysis based on BPPCT034 and CPSCT038 indicates two groups in dark yellow and green.

In the case of sour cherry, two distinct groups were observed, with two groups separated by small and large fruit, as shown in Figure 10. Samples divided into two groups showed a clear separation at 5 g in weight. One group, consisting of accessions with mostly large fruits, carried alleles CPSCT038_185_ and CPSCT038_204_ in two to four copies, while the group representing small-fruited accessions carried the allele CPSCT08_190_ in two to four copies. Regarding the BBPCT034 marker, the 204 bp allele in two copies was more common among the group of large-fruited sour cherries, whilst the 208 bp allele in two copies was more common in the group of small-fruited accessions.

## 3. Discussion

### 3.1. Sweet Cherry Analysis for Fruit Size and Weight

According to our findings, the Hungarian sweet cherry population and genebank accessions can be differentiated for fruit size and weight with the CPSCT038 and BPPCT034 SSR markers. In line with our previous findings, haplotype 190–255 has been observed in association with large fruits [19,25,31]. Haplotypes 192–223 and 204–235 were associated with smaller fruit, in contrast with previous findings from Zhang and colleagues [31], but in accordance with Rosyara and colleagues [19]. Allelic combination 190–257, however, was not observed in clear correlation with small fruit size, as has been suggested in previous research [32]. The differences in correlation could be explained by the fact that the Hungarian population did not clearly correlate with small fruit size and the indicated haplotype 190–257, suggesting that there is no connection between the haplotype 190–257 and small fruit size, but rather with medium to large. In our analysis, the largest and heaviest fruit were from ‘Vaszilisza’, with more than 10 g observed, which carried the haplotype 190–257. As previously published [32], the assignment of haplotype 190–257 to presumed small fruit size was due to the fact that this specific haplotype was rarely found in the assessed germplasm, with presumed selection against it during breeding; in contrast, we could detect it in several accessions. With a frequency of 0.16, allele BPPCT034_257_ was the third most common allele of this marker among our studied sweet cherries. This might suggest a distinct genetic background of our germplasm collection compared to cultivars analyzed by RosBREED [32]. Furthermore, when we observed individual alleles such as BPPCT034_235_, it indicated a weak negative correlation with fruit size and weight. CPSCT038_192_ and BPPCT034_223_ showed a moderate negative correlation with fruit characteristics. CPSCT038_190_ and BPPCT034_255_ both showed moderate-to-strong positive correlations with fruit size and weight characteristics, suggesting the importance of these alleles’ presence in future breeding selection.

The PCA showed distinct groups based on kinship, and these groups could be identified on the cladogram based on genetic distance. Since the evaluated sweet cherry accessions are mostly local landraces with unknown pedigree, when interpreting the cladogram, we have to rely on the names of the accessions. The cluster with large-fruited accessions having the 190–255 haplotype, and which are homozygous for this locus, contains two accessions with Ukrainian origin (‘Doncsanka’ and ‘Plakucsnaja’), as well as several possible variants of ‘Badacsonyi’ and ‘Kecskecsöcsű’. This suggests the possible relatedness of these accessions; however, ‘Badacsonyi óriás’ and ‘Kecskecsöcsű (II 7/41)’ are clustered in a different group. The same possible haplotype is shared by ‘Sam’ [32], the Hungarian commercial cultivars ‘Germersdorfi óriás’, ‘Katalin‘, ‘Vera’ and ‘Carmen’, as well as the Ukrainian ‘Krupnoplodnaya’ [25]. The ‘Badacsonyi’ landrace is regarded as a seedling of the cultivar ‘Germersdorfi óriás’, based on phenotypic similarities. This assumption might be correct, as they share the same genotype regarding the studied locus.

### 3.2. Sour Cherry Analysis for Fruit Size and Weight

In sour cherry, BPPCT034 and CPSCT038 SSR markers were tested to determine if they contributed to fruit size and weight in the Hungarian local germplasm. Allelic combinations were observed to be unique for most of the analyzed sour cherry population due to the high level of polymorphism. Only in a few cases was it possible to observe commonality in allelic combinations, with a small (190–190–190–190–208–208–226–230), medium (185–185–204–204–204–204–226–237; 185–185–204–204–226–226–237–237; 190–190–204–204–204–204–226–237) and large (185–185–185–185–204–204–226–237) fruit size and weight allelic combination found in more than one accession. However, due to high polymorphism, it seems that no clear allelic combination has been observed in correlation with fruit size, since many combinations are possible between alleles, and possible haplotypes consisted of multiple unique combinations. Increasing the population size and assessing parental lines could help to clarify which possible haplotype might correlate with large or small fruit size. The latter is not possible in our case due to the fact that many of the accessions analyzed are collected landraces with unknown ancestors, or cultivars obtained from open pollination.

Further correlation analysis of single alleles suggests a moderate-to-strong positive correlation between fruit size characteristics and the BPPCT034_204_ allele and a weak positive correlation with the BPPCT034_237_ allele, while BPPCT034_208_ showed a strong correlation with small fruit size. CPSCT038_185_ and CPSCT038_204_ were both moderately correlated with large fruits, while a weak to moderate negative correlation was observed between fruit size characteristics and CPSCT038_190_. Further investigations should be conducted to determine whether haplotypes with 185 and 204 alleles of CPSCT038 and 204 and 237 alleles of BBPCT034 could be considered possible carriers of large fruit, while haplotypes with CPSCT038_190_ or BPPCT034_208_ alleles could be considered carriers of small fruit types.

In contrast with our sweet cherry results, it does not appear that accessions containing the CPSCT038_190_ allele correlate with large fruit; instead, they have a weak negative effect on fruit size. This could be explained by the difference in fruit species and ploidy level observed in sweet and sour cherry, as well as possible insertion or deletion elements present in sour cherry.

In sour cherry, the PCA plot showed two distinct groups, and the same pattern could be observed in the cladogram. Similar to sweet cherry, the assessed accessions are mostly collected landraces with unknown ancestors, and some cultivars were obtained through open pollination. When analyzing their genetic relatedness, we must rely on the names of the accessions. All the ‘Pándy’-type sour cherries are found among the group of large-fruited accessions, including the Hungarian commercial cultivars ‘Favorit’ and ‘Érdi Jubileum’, both of which have ‘Pándy’ ancestors. However, the most important Hungarian commercial cultivar ‘Érdi bőtermő’ (‘Pándy’ × ’Neue Englische Wichsel’) clusters in another group. Several ’Cigánymeggy’-type accessions were analyzed in the study; they clustered in both groups, suggesting different genetic backgrounds.

## 4. Materials and Methods

### 4.1. Sample Collection and Phenotyping for Sweet and Sour Cherry

In the germplasm collection, 2 to 4 clones of the same accession were taken into account for our analysis, depending on the availability and presence of clones. Plant material was selected based on several years of phenotyping and the differences between accessions. Sweet cherry fruits from 40 individual accessions, and from the reference varieties ‘Van’ and ‘Regina’, were collected from individual trees for 3 to 5 years, between 2016 and 2021. For sour cherry, 31 accessions and reference varieties were selected, and phenotyping was performed between 2021 and 2023. The reference varieties (‘Érdi bőtermő’, ‘Kántorjánosi 3’ and ‘Újfehértói fürtös’—known under the cultivar name ‘Balaton’ in the USA) were included to obtain comparable and reproducible data and to compare these varieties with local accessions. Sample collection was performed during the maturation period, which varied each year, from around the middle of May to mid-June for sweet cherry and from the middle of June to the beginning of July for sour cherry. To ensure even distribution, around 500 g of fruits were collected from the north, south, east, and west orientations for each accession, and the fruits were picked randomly. For each accession analyzed, 20 fruits were selected for phenotyping based on their appearance, discarding fruits that appeared damaged. Sweet and sour cherry fruits were measured with a standard caliper (<0.1 mm) (Digital ABS Caliper, Mitutoyo Inc., Kawaski, Japan) for the diameter, length and thickness of the fruit, while a scientific precision scale (<0.01 g) (Kern & Sohn GmbH, Balingen, Germany) was used to measure fruit weight. For each accession, twenty healthy fruits were measured, and the measurements were repeated for each year of the analysis.

### 4.2. Genotyping: DNA Extraction, PCR, and Fragment Analysis

Four leaves from individual trees were collected for each accession of sweet and sour cherry. DNA was extracted following the manufacturer’s instructions using the Plant Genomic DNA Mini extraction kit (Viogene BioTek Corp., New Taipei City, Taiwan). An average concentration of 50 ng/µL was quantified using a microvolume spectrophotometer, Denovix DS-11 (DeNovix Inc., Wilmington, DE, USA). The polymerase chain reaction was set up with DreamTaq polymerase (5 U/µL; Thermo Scientific Inc., Waltham, MA, USA), 2.5 mM MgCl_2_, 0.2 mM of each dNTP, 0.25 µM of each primer and 1 ng of genomic DNA in a total volume of 20 µL per reaction. To decrease protein contamination, 45 mg/mL bovine serum albumin (BSA) and 90 mg/mL dimethylsulfoxide (DMSO) were also added to the reaction mix. PCR conditions were optimized according to the manufacturer’s protocol as follows: initial denaturation at 95 °C for 3 min followed by 35 cycles at 95 °C for 45 s, 50 °C and 56 °C for CPSCT038 and BPPCT034, respectively, each for 45 s, 72 °C for 45 s, with a final extension at 72 °C for 8 min. To assess the exact size of the SSR fragment, a fluorescent dye, 6-FAM, was added at the 5′ end of each forward primer, and samples were analyzed by capillary electrophoresis using an automated sequencer, ABI Prism 3100 Genetic analyzer (Applied Biosystems, Budapest, Hungary). Allele sizing and assessment were performed using Thermo Fisher Peak Scanner^TM^ software, available on the Thermo Fisher cloud platform (Applied Biosystems™, Thermo Fisher, Waltham, MA, USA). The primers selected were CPSCT038 [26] and BPPCT034 [33], and they were used in this analysis since they had already been tested for Hungarian sweet cherry elite cultivars [25].

### 4.3. Statistical Analysis

Statistical analysis was performed to differentiate the studied accessions and genotypes based on the assessed traits. Fruit weight was strongly correlated with diameter, length and thickness (Pearson’s r(3066) > 0.9, *p* < 0.001 for each trait in sweet cherry; r(1748) > 0.84, *p* < 0.001 for each trait in sour cherry), indicating high multicollinearity among size-related traits. Therefore, to avoid multicollinearity and to simplify interpretation, subsequent phenotype analyses focused on fruit weight, and one-way ANOVA was performed followed by the Games–Howell post hoc test to differentiate genotypes. The Games–Howell test was chosen to compare group means because Levene’s test was significant for the dependent variable fruit weight, indicating a violation of the homogeneity of variances assumption (F(5, 3062) = 141.79, *p* < 0.001; F(7, 1742) = 41.14, *p* < 0.01) in sweet and sour cherry, respectively. Continuing with the assumption checks, normality of the residuals was analyzed by the Shapiro–Wilk test (sweet cherry: W = 0.098, *p* < 0.001; sour cherry: W = 0.99, *p* < 0.001). Although the Shapiro–Wilk test was significant, we assessed normality of the data by visual inspection of histograms, as well as by skewness and kurtosis values (|skewness| < 1, |kurtosis| < 1), which indicated only minor deviations from normality. The data were therefore considered approximately normally distributed [34].

For genotype–phenotype correlation analysis, Pearson’s correlation was applied to detect correlations between individual alleles and the observed phenotypes. For each trait (in both sweet and sour cherry), normality was assessed using the Shapiro–Wilk test. Although it was significant (*p* < 0.05), skewness and kurtosis values were below 1 in absolute value, indicating only minor deviations from normality. Visual inspection of histograms and Q–Q plots also confirmed this. Therefore, the data were treated as approximately normally distributed, and Pearson’s correlation was applied.

One-way ANOVA and Pearson’s correlation were conducted using the software IBM SPSS Statistics for Windows version 26 [35].

Principal component analysis (PCA) was performed in RStudio [36] to differentiate between the accessions and the molecular markers in order to classify each accession based on the presence and distribution of each allele. Ploidy analysis was performed for CPSCT038 and BPPCT034 for sweet and sour cherries, where, in the RStudio script, data were transformed into vectors for allele analysis. After transformation, allele frequencies, expected heterozygosity (H_e_), polymorphism information content (PIC), effective multiplex ratio (EMR), marker index (MI), observed heterozygosity (H_o_), resolving power (RP) and discriminating power (DP) were analyzed based on previously published formulas [37,38].

## 5. Conclusions

Since the first analysis of the *FW_G2a* locus and the *PavCNR12* gene, several studies have been conducted on the importance of this locus and the correlation with fruit size and weight characteristics, and the flanking of the aforementioned gene by two molecular markers, BPPCT034 and CPSCT038 [19]. In a previous study, we tested both markers and their effect on fruit weight in commercial Hungarian sweet cherry cultivars [25]. In this study, we presented a larger collection of sweet cherry and also integrated sour cherry, demonstrating that SSR markers BPPCT034 and CPSCT038 can be used to screen Hungarian sweet and sour cherry local accessions.

Fruit size and weight are valuable characteristics for breeders, and the use of molecular markers as an integrating tool to screen populations for selection purposes is becoming more common, to be able to breed new varieties more efficiently. With marker-assisted selection (MAS), early selection of hybrids can be performed. Hybrids carrying alleles associated with big fruit size can be pre-selected, and a negative selection is also possible for potentially small-fruited hybrids at the seedling stage. Consequently, fewer hybrids need to be raised and evaluated over multiple years, reducing breeding costs and labor. Furthermore, careful selection of parental lines based on their genotype, rather than relying only on phenotypic observations, can increase the potential success rate in finding the hybrid with the desired trait. By screening parental cultivars and selecting those carrying alleles associated with big fruit size in homozygous form, the likelihood of large fruit size in the F1 population can be increased.

The germplasm material available in the collection gives us an advantageous availability of genetic variability, making it possible to identify possible candidates to be integrated into our breeding program. In our study, we focused on Hungarian local germplasm material to identify valuable accessions based on their fruit size and weight characteristics. In sweet cherry, the largest fruits were observed in the Ukrainian-derived accession ‘Vaszilisza’, whereas in sour cherry the largest fruits were recorded for ‘Mogyoródi kései’. Large fruits are very appreciated in local and international markets, especially when other characteristics such as fruit firmness, resistance to cracking and excellent fruit flavor profile are present as well [9,39,40]. The ‘Vaszilisza’ and ‘Mogyoródi kései’ accessions could be used in the future for their favorable characteristics and integrated into our breeding program for the development of new and unique Hungarian sweet and sour cherry varieties.

Several sweet cherry accessions were carrying the haplotype 190–255 (associated with large fruit size) in homozygous form. These accessions could be useful in breeding programs as parental lines, in which case the progeny would inherit this positive trait.

Molecular markers BPPCT034 and CPSCT038 both served as fruit size and weight indicators, being able to identify different accessions based on their fruit size. Furthermore, a significant correlation was observed with fruit size characteristics as well, in line with weight observations from our previous study in sweet cherry [25].

To our knowledge, this is the first study that included fruit size analysis of CPSCT038 and BPPCT034 markers in the sour cherry local population in Hungary.

## Figures and Tables

**Table 1:** BPPCT034 and CPSCT038 allelic frequencies in sweet and sour cherry observed in our study. The number of alleles is indicated for both sour and sweet cherry. Expected (H_e_) and observed heterozygosity (H_o_) are indicated. PIC: Polymorphism information content; EMR: Effective multiplex ratio; MI: Marker index; DP: Discriminating power; RP: Resolving power.

Sample	Marker	Allele N	H_e_	H_o_	PIC	EMR	MI	DP	RP	Frequency
Sour cherry	BPPCT034	10	0.99	0.94	0.05	29	1.53	0.99	3.48	*204:0.25; 208:0.13; 216:0.01; 218:0.04; 222:0.01; 224:0.02; 226:0.24; 230:0.07; 237:0.21; 251:0.02*
	CPSCT038	3	0.96	0.61	0.15	19	2.81	0.90	2.58	*185:0.31; 190:0.39; 204:0.31*
Sweet cherry	BPPCT034	9	0.75	0.45	0.07	18	1.34	0.90	2.80	*222:0.01; 223:0.24; 225:0.01; 229:0.01; 235:0.14; 237:0.02; 243:0.01; 255:0.39; 257:0.16*
	CPSCT038	4	0.52	0.33	0.08	13	1.03	0.73	1.55	*190:0.66; 192:0.15; 202:0.05; 204:0.14*

## Data Availability

The raw data supporting the conclusions of this article will be made available by the authors upon request.

## Supplementary Materials

The following supporting information can be downloaded at: https://www.mdpi.com/article/10.3390/plants15060856/s1, Table S1: Phenotype and haplotype observed in sweet cherry. Fruit measurements such as weight, diameter, length, and thickness were observed between 2016 and 2021. Mean values are shown for each trait. Allelic combinations assigned with different lowercase letters are significantly different based on mean fruit weight (*p* < 0.05); Table S2: Phenotype and haplotype observed in sour cherry. Fruit measurements such as weight, diameter, length, and thickness were observed between 2021 and 2023. Mean values are shown for each trait. Genotypes assigned with different lowercase letters are significantly different based on mean fruit weight (*p* < 0.05); Table S3: Pearson correlation for CPSCT038 and BPPCT034 alleles observed in sweet cherry and their phenotypes. Significance level is indicated as *p* < 0.05 (*) and *p* < 0.01 (**). Green values indicate positive correlation, while red values indicate negative correlation; Table S4: Pearson correlation for CPSCT038 and BPPCT034 alleles observed in sour cherry and their phenotypes. Significance level is indicated as *p* < 0.05 (*) and *p* < 0.01 (**). Green values indicate positive correlation, while red values indicate negative correlation.
